# Instrumental Analysis or Human Evaluation to Measure the Appearance, Smell, Flavor, and Physical Properties of Food

**DOI:** 10.3390/foods12183453

**Published:** 2023-09-15

**Authors:** Mohammed Saleh, Youngseung Lee

**Affiliations:** 1 Department of Nutrition and Food Technology, The University of Jordan, Amman 11942, Jordan; misaleh@ju.edu.jo; 2 Department of Food Science and Nutrition, Dankook University, Cheonan 31116, Republic of Korea

## 1. Introduction

Instrumental analysis and sensory evaluation are two fundamental approaches used to assess the quality of food products, encompassing attributes such as appearance, smell, flavor, and physical properties [1]. These approaches play distinct yet complementary roles in ensuring that food products not only meet safety standards but also cater to consumer preferences. Instrumental analysis involves the utilization of scientific instruments and techniques to acquire objective and quantifiable data regarding various food properties. These properties encompass chemical composition, physical characteristics, and structural attributes. However, instrumental analysis may not fully capture the sensory perception of food.

In contrast, sensory evaluation relies on human sensory perception to assess food product attributes. It employs either trained panelists or untrained consumers to provide feedback on taste, aroma, texture, appearance, and overall liking. While sensory evaluation offers a holistic understanding of how individuals perceive food, it can be influenced by subjectivity. Together, these two approaches form a powerful combination. Instrumental analysis helps quantify specific characteristics of food, offering precise data. In parallel, sensory evaluation provides insights into how consumers perceive and experience these characteristics. This dual assessment ensures that food products not only adhere to safety and regulatory standards but also align with consumer preferences and expectations.

The correlation between instrumental analysis and sensory evaluation is pivotal in predicting the quality characteristics of foods. While instrumental analysis supplies objective data, sensory evaluation bridges the gap between the technical aspects of food and how consumers perceive and experience it. Understanding the correlation between these two approaches empowers food scientists and manufacturers to produce high-quality products that resonate with consumer preferences. This synergy between instrumental analysis and sensory evaluation enhances our understanding of food quality and appeal, ultimately benefiting both producers and consumers in the market.

## 2. Instrumental Analysis to Predict the Quality Characteristics of Food

Instrumental analysis plays a pivotal role in predicting the quality characteristics of food products. Food quality encompasses a broad spectrum of attributes, including chemical composition, physical properties, safety, and sensory characteristics. Various instrumental methods are employed in this process, including HPLC, GC-MS, FTIR, and texture analysis using specialized texture analyzers. Additionally, cutting-edge technologies such as electronic eyes, electronic tongues, and electronic noses have gained prominence in determining the taste, aroma, and flavor profiles of food products.

Texture analyzers, for instance, are instrumental in assessing the mechanical properties of food, allowing the prediction of attributes such as hardness, springiness, chewiness, and crispness of food products. These characteristics closely mimic human mastication and are vital for predicting the mouthfeel and texture-related quality of foods.

In the realm of color assessment, spectrophotometers and colorimeters are invaluable tools, providing objective measurements of color attributes. They significantly aid in predicting the appearance and visual appeal of food products. Notably, electronic eyes (EE) have emerged as a promising technology for objectively measuring various colors in food. EE is a specialized instrument designed to replicate human visual interpretation, capturing data related to color and appearance characteristics in samples. It enables impartial assessments of an item’s color attributes, with EE sensors employing colorimetry, spectrophotometry, or computer vision as their core technologies [2].

Moreover, the combination of GC-MS and electronic noses can effectively analyze volatile compounds, predicting the flavor and aroma profiles of food products. This capability is particularly valuable in the development of flavors and fragrances. Electronic noses (ENs) are distinct analytical instruments consisting of arrays of electronic chemical sensors, each with limited specificity. Coupled with a sophisticated pattern recognition system, ENs have a unique capability: they can recognize complete volatile aroma mixtures, whether simple or complex, without the need to identify individual chemical components within the sample mixture [2].

On a related note, electronic tongues (ETs) serve as multisensory systems tailored for liquid analysis. They utilize arrays of chemical sensors in conjunction with pattern recognition systems. While sharing the fundamental principle of integrating data from nonspecific sensors with a pattern recognition system, ETs differentiate themselves by specializing in the analysis of liquid samples, setting them apart from ENs.

## 3. Sensory Evaluation to Predict the Quality Characteristics of Food

Sensory evaluation is a vital tool for understanding product quality and consumer preferences. Traditionally, it has been divided into two main domains: analytical tests that objectively measure the intensity of sensory attributes, and tests that assess consumer acceptability [3].

Within the realm of analytical tests, descriptive analysis conducted by trained panelists has played a crucial role in ensuring food product quality and driving innovation in the food industry. However, it has its limitations. Trained panelists, while highly skilled, may not accurately gauge a product’s hedonic appeal since their training focuses on specific criteria, which may not always align with individual preferences [4]. Moreover, a small group of trained panelists cannot fully represent the entire target market or predict how a product will be received in the broader market. On the contrary, consumers have often been deemed unsuitable for analytical tasks due to their lack of formal training in quantifying sensory characteristics. Nevertheless, sensory evaluation remains indispensable for comprehending consumer preferences, optimizing product formulations, and ensuring that food products meet sensory expectations.

Despite its significance, sensory evaluation has several limitations and challenges, as follows. First, sensory evaluation relies on human perception, which is inherently subjective. Different individuals may perceive and describe sensory attributes differently, leading to variability in results. Second, even trained sensory panels can exhibit variations in their assessments influenced by factors such as differences in sensory acuity, fatigue, mood, and inconsistencies in sample preparation. Third, panelists may experience sensory fatigue during extended tasting sessions, which can reduce their sensitivity and evaluation accuracy. Fourth, conducting sensory evaluations can be resource-intensive, requiring time, specialized facilities, and trained personnel. This resource demand may limit its feasibility for some companies or research projects. In summary, while sensory evaluation provides valuable insights into product quality and consumer preferences, it is essential to be mindful of its inherent subjectivity and the need for proper training and controls to mitigate potential limitations and challenges.

## 4. Correlation between Instrumental Analysis and Sensory Evaluation to Predict the Quality Characteristics of Food

To address the limitations of sensory evaluation, researchers and industries often combine sensory evaluation with instrumental analysis and consumer testing. This holistic approach provides a more comprehensive understanding of product quality and consumer preferences in the realm of food science and technology. The correlation between instrumental analysis and sensory evaluation is a fundamental aspect of this approach, offering complementary insights into food products.

Researchers frequently conduct correlation studies to assess how well instrumental measurements align with sensory perceptions [5]. For example, they may investigate the relationship between instrumental texture measurements and sensory evaluations of texture. These studies aim to pinpoint the instrumental parameters most relevant to sensory attributes and overall product quality. These correlations between instrumental data and sensory scores serve as valuable tools for manufacturers to uphold consistent product quality. When instrumental measurements deviate from acceptable sensory standards, necessary adjustments can be made in the production process. Moreover, the correlation between instrumental and sensory data aids in uncovering the reasons behind consumer preferences for certain products. It can reveal that specific attributes, such as texture or aroma components, significantly contribute to higher liking scores.

The correlation data also empowers researchers and food developers to optimize product formulations and processing methods. This optimization process aims to achieve desired sensory attributes while simultaneously meeting safety and regulatory requirements. Additionally, in the realm of food research and innovation, instrumental analysis sheds light on the underlying chemical and physical changes that occur during food processing or storage. Sensory evaluation acts as a crucial validation tool by assessing how these changes impact consumer perception.
